# Weekly Iron-Folic Acid Supplementation with Regular Deworming Is Cost-Effective in Preventing Anaemia in Women of Reproductive Age in Vietnam

**DOI:** 10.1371/journal.pone.0023723

**Published:** 2011-09-08

**Authors:** Gerard J. Casey, Davide Sartori, Susan E. Horton, Tran Q. Phuc, Luong B. Phu, Dang T. Thach, Tran C. Dai, Giovanni Fattore, Antonio Montresor, Beverley-A. Biggs

**Affiliations:** 1 Department of Medicine (RMH/WH), The University of Melbourne, The Royal Melbourne Hospital, Parkville, Australia; 2 Università Commerciale Luigi Bocconi, Milano, Italy; 3 Balsillie School of International Affairs, University of Waterloo, Ontario, Canada; 4 National Institute of Malariology, Parasitology and Entomology, Tu Liem, Hanoi, Vietnam; 5 Yen Bai Medical College, Yen Bai, Viet Nam; 6 World Health Organization, Hanoi, Vietnam; 7 Bocconi University, Milano, Italy; 8 World Health Organization, Geneva, Switzerland; 9 Centre of Clinical Research Excellence in Infectious Diseases (CCREID), The Royal Melbourne Hospital, Parkville, Australia; University of California Los Angeles, United States of America

## Abstract

**Background:**

To estimate the cost and cost-effectiveness of a project administering de-worming and weekly iron-folic acid supplementation to control anaemia in women of reproductive age in Yen Bai province, Vietnam.

**Methods and Findings:**

Cost effectiveness was evaluated using data on programmatic costs based on two surveys in 2006 and 2009 and impact on anaemia and iron status collected in 2006, 2007, and 2008. Data on initial costs for training and educational materials were obtained from the records of the National Institute of Malariology, Parasitology and Entomology and the Yen Bai Malaria Control Program. Structured questionnaires for health workers at district, commune and village level were used to collect ongoing distribution and monitoring costs, and for participants to collect transport and loss of earnings costs. The cost per woman treated (defined as consuming at least 75% of the recommended intake) was USD0.76 per annum. This estimate includes financial costs (for supplies, training), and costs of health care workers' time. Prevalence of anaemia fell from 38% at baseline, to 20% after 12 months. Thus, the cost-effectiveness of the project is assessed at USD 4.24 per anaemia case prevented per year. Based on estimated productivity gains for adult women, the benefit:cost ratio is 6.7∶1. Cost of the supplements and anthelminthics was 47% of the total, while costs of training, monitoring, and health workers' time accounted for 53%.

**Conclusion:**

The study shows that weekly iron-folic acid supplementation and regular de-worming is a low-cost and cost-effective intervention and would be appropriate for population-based introduction in settings with a high prevalence of anaemia and iron deficiency and low malaria infection rates.

## Introduction

WHO estimates suggest that globally up to 500 million women of reproductive age suffer from anaemia. Women and children are most vulnerable due to greater iron requirements and those in resource constrained countries are particularly at risk [1]. Severe maternal anaemia increases the risk of maternal and neonatal mortality and morbidity while less severe anaemia and iron deficiency have been linked to sub-optimal fetal growth, premature birth and low birth weight, as well as iron deficiency in infants [2]–[4] which may impair cognitive development [5]. On a broader scale the reduced capacity for work and education due to anaemia and iron deficiency can adversely impact a population's social and economic development [6].

WHO guidelines recommend daily iron supplementation for pregnant women and children 6–24 months where anaemia rates are 40% or more [7]. A recent international expert consultation organized by WHO went further and recommended weekly supplementation with iron and folic acid for all non-pregnant women of reproductive age in areas where anaemia prevalence is greater than 20% [8]. A systematic review of 25 original studies concluded that this approach of weekly iron and folic acid supplementation was safe and also effective in improving haemoglobin concentration in women of reproductive age [9]. However, there is no data on cost and cost-effectiveness of population-based weekly supplementation programs, and limited data on other nutritional supplementation programs.

In Vietnam, there has been an increasing awareness of nutritional deficiencies and anaemia, particularly in rural populations. A national survey in 2000 found anaemia rates were 24.3% for non-pregnant women nationally (26.3% in rural areas), and 32.2% in pregnant women (33.8% in rural areas) [10]. Contributing factors included lack of variety in the diet associated with low income, compounded by high rates of infection with soil-transmitted helminths, particularly hookworm. A study of pregnant women in the rural central highlands in 2001 identified anaemia prevalence in pregnant women, post-partum women and non-pregnant women of 53%, 62% and 54% respectively [11].

As a consequence the Vietnam National Nutrition Strategy 2011–2020 has the reduction of iron deficiency anemia in children less than 5 years of age and reproductive-age women as an objective. However the costs of potential strategies for achieving this are currently unknown.

In 2006, following a baseline study of the prevalence of anaemia, iron deficiency and soil transmitted helminth infection prevalences [12], we introduced an anaemia and helminth control program consisting of weekly iron-folic acid supplementation and regular deworming for women of reproductive age in Yen Bai, a rural province in the northern mountainous region of Vietnam. Surveys were conducted to assess program impact, compliance and cost [13], [14]. We report here the cost and cost-effectiveness of the de-worming and weekly iron-folic acid intervention, which has now been delivered to women in Yen Bai province since May 2006.

## Materials and Methods

### Program implementation, compliance and impact assessment

In May 2006 the National Institute of Malariology, Parasitology and Entomology (NIMPE) and partners introduced a pilot program consisting of weekly iron-folic acid supplementation and periodic de-worming for women of reproductive age in Yen Bai province. Yen Bai is a mountainous, predominantly rural province located approximately 180 km from Hanoi. The intervention was implemented in two districts targeting all 50,000 women of reproductive age, and a cost analysis was undertaken.

The integration of the weekly iron-folic acid supplement and deworming program into the existing health infrastructure in Yen Bai province has been described elsewhere [14]. In brief, community leaders and health staff at all levels were sensitized, promotional materials were developed, and village health workers were trained to distribute supplements each month to non-pregnant women. Pregnant women were advised to use the daily supplements provided through Government antenatal clinics, or if these were not available, to use a double dose of the weekly supplements. Non-pregnant women were given supervised doses of albendazole (400 mg) every four months in the first year of the program and every six months thereafter.

The impact of the program on women's iron and helminth infection status in the first 12 months was assessed using structured questionnaires, stool examination and haemoglobin assessment at the field site, and blood sample analysis for ferritin, soluble Transferrin Receptor and C-Reactive Protein (to identify the presence of acute infection). These surveys were conducted at 3 and 12 months post-implementation [15].

With evidence of the program's positive impact on women's iron status the weekly supplement program with twice-yearly de-worming was scaled up to province level (with an estimated target group of 250,000 women of reproductive age) commencing March 2008. A follow-up survey was repeated at 30 months post-implementation.

The effectiveness of the distribution system and women's compliance were assessed by an independent Non-Government Organization at 10 weeks and 18 and 36 months post-implementation. Structured questionnaires were administered to assess the availability of supplements at each level of the distribution chain and women's attitude about, and their history of, taking the supplements [13].

### Program cost assessment

Costs of the intervention included both programmatic costs and health system costs. These costs were identified through the accounts of the relevant agencies and structured interviews with health staff at each level of the distribution chain. In addition, participating women were surveyed to assess their estimate of costs involved in accessing the supplements. Health staff interviews and participating women surveys were conducted in 2006 and 2009.

Programmatic costs included cost of supplements, which were originally distributed in tamper-proof bottles (UNICEF, Copenhagen), and after 12 months sourced domestically in blister packs consisting of 6 tear-off strips of 5 tablets, each strip enough for one month (Nam Ha Pharmaceutical Company, Nam Dinh, Vietnam). The albendazole for helminth control was kindly donated by WHO. In 2006 Albendazole sourced from UNICEF was quoted at USD1.617/100 tabs. *Distribution/storage costs* were for receiving the supplements at NIMPE and then periodically transporting them to Yen Bai city. After 12 months the cost of delivery to the province was included in the contract with the manufacturer. Once there, existing distribution channels of the public health care system were utilized. *Promotional and educational material costs* included the development and production of posters and flip-charts, which were then disseminated via the public health system. *Training* of trainers involved national staff training staff from the provincial level, who then trained district, commune and village level health workers. *Monitoring* involved printing forms, distributing them to the relevant level (village level for iron supplements: commune level for de-worming), and then returning them to Yen Bai and monthly mailing back to NIMPE. All these costs could be identified from financial records. *Amortization* of training and promotional material production costs was assumed to be over 5 years. The discount rate assumed was zero: using a higher discount rate would increase costs by a trivial amount.

Health system costs included institutional administrative costs and health worker income. Personnel costs were imputed based on information obtained from interviews with district, commune and village health workers about their total salary and the proportion of time they spent on this intervention. No monetary value was assigned to the women's time since the time required per person was very modest and respondents stated that this did not involve loss of work time or incur a financial cost.

Costs resulting from the research activities on program impact and compliance rates are not included. All cost data were collected in Viet Nam dong and converted to US dollars using the exchange rate on March 10 2010 (USD1.00 =  VND18,685).

### Benefit-Cost Analysis

Effectiveness data were obtained from the two pilot districts, which were surveyed at baseline and at 3, 12 and 30 months post-implementation for the impact on anaemia and helminth infection status, and independent compliance monitoring at 10 weeks, 18 and 36 months post-implementation. The treatment cost per woman was calculated based on total cost divided by the number of women taking 75% or more of the supplements. The cost per anaemia case prevented was calculated as the total cost of the program (both set up and maintenance costs) divided by the difference between the total number of anaemia cases at baseline and the number of anaemia cases after 12 months of implementation. The benefit:cost ratio was estimated using the methods described by Horton and Ross (2003) [6] based on improved labour market productivity for women of reproductive age after removal from anaemia.

## Results

The basis and source of data for programmatic and health system costs are shown in Table 1. Promotional and training costs are amortized over 5 years as a realistic assumption of the lifespan of printed materials and accounting for staff turnover in the health care system.

**Table 1:** Basis for costs and source of data.

**Project costs**	
*Iron-folic acid* *supplements* *Promotion* *Training* *Monitoring*	USD4.55/′000 tablets: 52 tablets/year, target population 250,000; costs, insurance, freight included. Source Project documentation, University of Melbourne/NIMPE. Distribution and storage USD3,597: source Yen Bai People's Committee financial recordsPromotional material development and production: amortized over 5 years. Source Project documentation, University of MelbourneTraining of trainers amortized over 5 years: USD85.67/year; training of local health staff USD10/person, 2000 staff, amortized over 5 years. Source Project documentation, University of Melbourne/NIMPE. Support provided by NIMPE: source NIMPE financial records
**Health care system costs**	
*National Institute* *District Health Service* *Commune Health Service* *Village Health Workers*	Source: NIMPE financial recordsAverage DHW income (USD260/month) X share of monthly hours worked on project (11%) X 3 workers/district X 7 districtsAverage CHW income (USD157/month) X share of monthly hours worked on project (10%) X 175 communesAverage VHW income (USD13/month) X share of monthly hours worked on project (30%) X 2000 individualsNon-salary costs are also incurred at each level (obtained from worker surveys)

CHW  =  commune health worker, DHW  =  district health worker, VHW  =  village health worker.

A breakdown of unit costs is shown in Table 2. The total annual cost of the program was USD 133,900 of which programmatic costs made up 69% of the total, the major component being the supply and transport of supplements (47% of the total). Health care system costs made up 31% of the total, the largest component of which was support for nearly 2000 village health workers.

**Table 2:** Annual cost of weekly iron supplementation, Yen Bai province, Vietnam.

**Project costs (USD)**	
*Iron-folic acid supplements (purchase, transport and storage)* *Promotion* *Training* *Monitoring*	62,7348144,08625,330
**Subtotal**	92,878
**Health care system costs (USD)**	
*National Institute* *District Health Service* *Commune Health Service* *Village Health Workers*	6084,4967,81228,020
**Total**	133,900
Number of women targetedNumber of women treated^1^	250,000175,581
Cost per woman treated	0.76

1 81% of target population accessed; compliance (taking > = 75% of recommended intake) was 87%[13].

The effective target group accessed was calculated at 70% of the 250,00 eligible women or 175,581 women of reproductive age. This was based on the independent monitoring survey one year after the province-wide expansion of the program, at which time 81% of surveyed women were receiving the supplements regularly and 87% of those were taking 75% or more of the supplied supplements [13].

As a result the calculated cost per woman treated is USD 0.76 per year (USD133900 / 175581).

The long-term impact of the Yen Bai program is shown in Table 3. After 12 months of the project mean hemoglobin increased from 122 g/L to 130g/L, the prevalence of anaemia decreased from 38% to 20% and the prevalence of hookworm infection decreased from 76% to 22% [13]. Assuming that anaemia cases prevented is the measure of effectiveness, the cost-effectiveness of the intervention is USD 4.24 per anaemia case prevented (USD133900 / (175581×18)). Rates of severe anemia in women of reproductive age fell from 1.1% to 0% after 3 months of the intervention [15]. We used calculations from Horton and Ross (2003) [6] to estimate a benefit:cost ratio based on improved labour market productivity for women of reproductive age. These authors estimate that the productivity gains for an individual removed from anaemia are 5% in manual occupations (70% of employment in a country such as Viet Nam), and 17% in heavy manual occupations (which form 12.7% of employment in manual occupations in Viet Nam: the share of agriculture in GDP is 22.1%) [16]. There are an estimated 22.2 m women of reproductive age (15–44) in Viet Nam out of a working-age population (15–64) of 60.6 m (and total population 89 m) [17]. Reducing anaemia rates by 18.2% for women of reproductive age therefore constitutes a 6.7% reduction in anaemia on average in the labour force (using the proportion of women of reproductive age in the overall labour force in Vietnam: women's labour force participation rate is similar to men's). The estimated dollar value per capita of the productivity gains are calculated as USD1.29, i.e. $5.18 per woman of reproductive age, as compared to the cost of supplementation per woman of reproductive age of USD0.76. Hence the benefit: cost ratio is approximately 6.7∶1, just based on improvements in productivity of women of reproductive age in the workforce. This means that for each dollar invested in a weekly iron supplementation and de-worming program the monetary value in terms of productivity was USD6.70. There may be additional financial benefits associated with reductions in other health care costs such as those associated with reduced maternal and infant mortality, and a reduction in neural tube defects which cannot readily be quantified. In addition there are possible effects on reduced anaemia in children and men associated with de-worming women of reproductive age and a consequent reduction in helminth transmission.

**Table 3:** Effectiveness of the Yen Bai iron supplementation and de-worming program.

Survey	Sample size	Haemoglobin (g/L)	Prevalence of anaemia
Baseline	349	122	38%
3 month	574	126	27%
12 month	572	131	20%
30 month	303	130	19%

Data sourced from Casey et al [13].

## Discussion

The provision of iron-folic acid supplements on a weekly basis for women of reproductive age is aimed at improving iron stores, particularly prior to pregnancy and during the first trimester before women access ante-natal care. Pre-pregnancy weekly supplements that contain folic acid may also decrease the risk of neural tube defects. A successful supplementation program must include training of health care system workers, provision of accessible educational materials for the target group and the broader community and a reliable distribution system. Therefore these costs need to be incorporated into the overall cost of an intervention program.

We have demonstrated the effective uptake of weekly iron-folic acid supplementation by 70% of woman in Yen Bai province with an annual cost of USD 0.76/woman. This compares to estimates by the National Institute of Nutrition that only 20% of pregnant women are covered by the national antenatal program of daily iron supplementation for which there is no costing. [18]. The costing reported here includes distribution of supplements, health staff training, production and distribution of educational materials and regular monitoring.

The possible reasons for the higher uptake of the weekly supplements are many. One likely reason for the difference is convenience. Antenatal supplements for pregnant women have to be accessed at the Commune Health Station, which is generally further for women to travel. Weekly supplements for women of reproductive age in the program reported here are accessed at village level. The 2009 survey identified that it took women an average of 17 minutes to access the distribution point for weekly supplements.

Another reason for high compliance with the weekly program was the maintenance of a reliable supply. A previous study has shown that a poor distribution system and irregular availability were the most significant environmental risk factors for taking iron supplementation continuously [19]. Also, weekly supplements may be better tolerated, with less frequent side-effects thereby increasing acceptance and compliance. The extensive training and community education program was another positive factor which increased acceptance of the supplements as a valuable contribution to women's health.

The main limitation of the impact evaluation was the lack of a control arm where no intervention was given. However, we considered it unethical to withhold iron-folic acid supplementation and deworming from women of reproductive age over a long period of time given the evidence from short-term studies on the efficacy of weekly iron-folic acid supplementation [8].

A previous study in Viet Nam followed women of reproductive age receiving weekly supplements, some of whom became pregnant at various points during the 12 months of follow-up. Those who had a longer period of weekly supplementation prior to pregnancy maintained higher Hb concentration during pregnancy [20]. Our study did not have a sample group large or accessible enough to allow an assessment of long-term pregnancy outcomes. With a larger sample, it would be possible to extend our calculations to estimate the cost per death averted with a reduction of the prevalence of severe anemia in pregnant women. The relative risk of mortality associated with a 10 g/L increase in hemoglobin is estimated as 0.75 for maternal mortality, and 0.72 for perinatal mortality [21].

The annual cost of USD0.76 per woman treated is consistent with a cost estimate of USD10-13 per pregnancy [22], [23], since pregnant women receive more tablets. A recent trial in northern China identified an annual cost of USD40.00 to improve antenatal, delivery and postnatal care of which 22% (USD8.80) was to supply and distribute daily supplements to pregnant women [24]. Data on cost per pregnancy of daily iron supplementation is quite weak. Baltussen et al (2004) [22] use the WHO “ingredients” approach to estimate costs (at 95% coverage) for various regions. For those countries in the Southeast Asia region (SearD) with high adult and child mortality rates, they estimate the cost as $10.12 (but as high as $46.04 per pregnancy in the low mortality countries in the Europe region, EurA). Behrman, Alderman and Hoddinott, 2004 [23], cite estimates for Nepal of $13.14 per pregnancy (based on program estimates extrapolated from a research study).

The estimated benefit:cost ratio of 6.7∶1 is similar to the median benefit:cost ratio for iron fortification estimated by Horton and Ross [6] for a range of countries. Supplementation of women is more costly per person, but has a stronger effect on anaemia. However, fortification benefits both men and women while supplementation directly benefits only women and will only have large labour market benefits in countries where women's participation in the labour market is high. The cost per woman is around four to five times higher than that of fortification programs. However, in Viet Nam fortification of cereals is not easily available for rural areas (rice, the main staple, is more costly to fortify, and less feasible to fortify given the lack of central processing). There have been experiments with fortification of fish sauce however this is not yet on a commercial scale. At present a supplementation program is the most feasible method for addressing iron and folate deficiencies in Viet Nam.

The cost of coverage with weekly iron-folic acid is comparable to program estimates for distribution of other nutritional supplements such as twice-yearly vitamin A, and zinc. A recent survey [25] of the cost of micronutrient supplementation programs (both published and “grey” literature) identified 43 studies, of which 31 were for vitamin A, and the rest for iron (7), iodine (4) and zinc (1). Mean cost per beneficiary were calculated as USD1.95 (vitamin A), USD3.64 (iodine), USD0.52 (zinc – only 1 program), but was not calculated for iron. Mean cost per person-year of “useful” coverage was estimated as USD8.53 (vitamin A) and USD0.74 (iodine) but not available for iron or zinc. Although Vitamin A capsules were less costly than iron per person (USD0.12/person/year compared to USD0.36), delivery costs for vitamin A were higher: USD1.83 compared to USD0.40. This may reflect lower coverage with volunteer health workers and lower population densities in Africa, where most costing studies of vitamin A were done.

The reported cost structures of delivery for these programs were however somewhat similar. Promotion accounted for 11% of the non-supplement costs, training 6% , volunteer time 24%, temporary paid workers 10%, permanent, salaried staff 41%, and other 11% for vitamin A. For the Yen Bai iron-folic acid supplementation program, promotion accounted for 1% of the non-supplement costs, training 6%, monitoring 36%, village health worker time 39%, and permanent, salaried staff 18%.

The costs of providing weekly iron-folic acid in a program setting may also be useful for estimating costs of providing micronutrient powders for home fortification for children 6–36 months, and for therapeutic zinc for children under five. Program cost data for these latter two interventions are almost non-existent.

This study therefore fills a major gap in the cost literature, namely estimating the cost of implementing weekly iron supplementation programs. It has policy significance given the recommendation to expand coverage of supplementation with weekly iron and folic acid supplements to women of reproductive age, as well as for other similar supplementation programs, such as multiple micronutrient powders and zinc for children.

## References

[1] McLean E, Cogswell M, Egli I, Wojdyla D, de Benoist B (2009). Worldwide prevalence of anaemia, WHO Vitamin and Mineral Nutrition Information System, 1993–2005.. Public Health Nutr.

[2] Allen LH (2000). Anemia and iron deficiency: effects on pregnancy outcome.. Am J Clin Nutr.

[3] Hindmarsh PC, Geary MP, Rodeck CH, Jackson MR, Kingdom JC (2000). Effect of early maternal iron stores on placental weight and structure.. Lancet.

[4] Scholl TO, Hediger ML, Fischer RL, Shearer JW (1992). Anemia vs iron deficiency: increased risk of preterm delivery in a prospective study.. Am J Clin Nutr.

[5] Preziosi P, Prual A, Galan P, Daouda H, Boureima H (1997). Effect of iron supplementation on the iron status of pregnant women: consequences for newborns.. Am J Clin Nutr.

[6] Horton S, Ross J (2003). The economics of iron deficiency. Food Policy..

[7] WHO (2001). Iron Deficiency Anaemia - Assessment, Prevention and Control.. A Guide for Programme Mmanagers. WHO/NHD/01.3 WHO. Geneva.

[8] WHO (2009). http://www.searo.who.int/LinkFiles/Nutrition_for_Health_and_Development_WHO_weekly_iron_folic_acid.pdf.

[9] Margetts BM (2007). Weekly iron and folic acid supplementation for women of reproductive age: effectiveness and safety..

[10] Khoi HH, Khan NC, Tam NC, Mai LB, Hao LQ (2001). Report on Vietnam National Anaemia Survey, 2000..

[11] Trinh LT, Dibley M (2007). Anaemia in pregnant, postpartum and non pregnant women in Lak district, Daklak province of Vietnam.. Asia Pac J Clin Nutr.

[12] Pasricha SR, Caruana SR, Phuc TQ, Casey GJ, Jolley D (2008). Anemia, iron deficiency, meat consumption, and hookworm infection in women of reproductive age in northwest Vietnam.. Am J Trop Med Hyg.

[13] Casey GJ, Jolley D, Phuc TQ, Tinh TT, Tho DH (2010). Long-term weekly iron-folic acid and de-worming is associated with stabilised haemoglobin and increasing iron stores in non-pregnant women in Vietnam.. PLoS ONE.

[14] Phuc T, Mihrshahi S, Casey G, Phu L, Tien N (2009). Lessons learned from implementation of a demonstration program to reduce the burden of anemia and hookworm in women in Yen Bai Province, Viet Nam.. BMC Public Health.

[15] Casey G, Phuc T, MacGregor L, Montresor A, Mihrshahi S et al (2009). A free weekly iron-folic acid supplementation and regular deworming program is associated with improved hemoglobin and iron status indicators in Vietnamese women.. BMC Public Health.

[16] World Bank (2010). World Development Indicators.. http://data.worldbank.org/indicator.

[17] UN (2008). Revision Population Database.. http://esa.un.org/unpp/index.asp?panel=2.

[18] Sartori DA (2007). Cost-effectiveness analysis of deworming and iron supplementation in Yen Bai, Vietnam..

[19] Aikawa R, Ngyen CK, Sasaki S, Binns CW (2006). Risk factors for iron-deficiency anaemia among pregnant women living in rural Vietnam.. Public Health Nutr.

[20] Berger J, Thanh HT, Cavalli-Sforza T, Smitasiri S, Khan NC (2005). Community mobilization and social marketing to promote weekly iron-folic acid supplementation in women of reproductive age in Vietnam: impact on anemia and iron status.. Nutr Rev.

[21] Stoltzfus RJ, Mullany L, Black RE, Ezzati M, Lopez AD, Rodgers A (2005). Iron deficiency anemia.. Comparative quantification of health risks: global and regional burden of disease attributable to selected major risk factors.

[22] Baltussen R, Knai C, Sharan M (2004). Iron fortification and iron supplementation are cost-effective interventions to reduce iron deficiency in four subregions of the world.. J Nutr.

[23] Behrman J, Alderman H, Hoddinott J, Lomborg G (2004). Hunger and Malnutrition.. Global Crises, Global Solutions.

[24] Zeng L, Yan H, Cheng Y, Dang S, Dibley MJ (2009). Adherence and costs of micronutrient supplementation in pregnancy in a double-blind, randomized, controlled trial in rural western China.. Food Nutr Bull.

[25] Fiedler JL, Sanghvi TG, Saunders MK (2008). A review of the micronutrient intervention cost literature: program design and policy lessons.. Int J Health Plann Manage.

